# Decline in Emotional Face Recognition Among Elderly People May Reflect Mild Cognitive Impairment

**DOI:** 10.3389/fpsyg.2021.664367

**Published:** 2021-06-07

**Authors:** Ryuta Ochi, Akira Midorikawa

**Affiliations:** ^1^Department of Psychology, Graduate School of Letters, Chuo University, Tokyo, Japan; ^2^Department of Psychology, Faculty of Letters, Chuo University, Tokyo, Japan

**Keywords:** emotional face recognition, elderly people, cognitive function, depression, multiple regression analyses

## Abstract

**Background:**

As with cognitive function, the ability to recognize emotions changes with age. In the literature regarding the relationship between recognition of emotion and cognitive function during aging, the effects of predictors such as aging, emotional state, and cognitive domains on emotion recognition are unclear. This study was performed to clarify the cognitive functions underlying recognition of emotional facial expressions, and to evaluate the effects of depressive mood on recognition of emotion in elderly subjects, as well as to reproduce the effects of aging on the recognition of emotional facial expressions.

**Materials and Methods:**

A total of 26 young (mean age = 20.9 years) and 30 elderly subjects (71.6 years) participated in the study. All subjects participated in face perception, face matching, emotion matching, and emotion selection tasks. In addition, elderly subjects were administered a multicomponent cognitive test: the Neurobehavioral Cognitive Status Examination (Cognistat) and the Geriatric Depression Scale-Short Version. We analyzed these factors using multiple linear regression.

**Results:**

There were no significant differences between the two groups in the face perception task, but in the face matching, emotion matching, and emotion selection tasks, elderly subjects showed significantly poorer performance. Among elderly subjects, multiple regression analyses showed that performance on the emotion matching task was predicted by age, emotional status, and cognitive function, but paradoxical relationships were observed between recognition of emotional faces and some verbal functions. In addition, 47% of elderly participants showed cognitive decline in one or more domains, although all of them had total Cognistat scores above the cutoff.

**Conclusion:**

It might be crucial to consider preclinical pathological changes such as mild cognitive impairment when testing for age effects in elderly populations.

## Introduction

As with cognitive functions, it is crucial for people to be able to recognize emotions. Therefore, there is a long history of research on emotion recognition in subjects ranging from infants to the elderly (Bridges, 1932; Gitelson, 1948). Some authors have suggested that the ability to recognize emotions changes with age (Isaacowitz et al., 2007; Mill et al., 2009). As age-related differences have been observed not only in emotion recognition but also in cognitive functions (Glisky, 2007), their relationship has been examined. There are two main views of the relationship between age-related changes in emotional and cognitive functions in the literature. One perspective suggests that changes in emotion recognition are independent of those in cognitive function, although both are influenced by age. For example, Baena et al. (2010), who examined younger and older adults using an emotional identification task and cognitive tests targeting prefrontal functions, suggested that age effects may contribute independently to each function. The second viewpoint suggests that changes in emotion recognition are dependent on cognitive functions. Suzuki and Akiyama (2013) examined cognitive and emotional function in younger and older adults and reported that age-related deficits in emotion identification could be explained by general cognitive abilities and not by age itself. More recently, Virtanen et al. (2017) focused on an elderly population using large-scale sampling and suggested that the decline in emotion recognition may depend on cognitive functions. In their study, declines in the recognition of some types of emotion, such as anger, fear, and disgust, were sensitive to cognitive deterioration even when cognitive function was within the normal range, i.e., Mini-Mental State Examination (MMSE) scores were above the cutoff point. As their study adjusted for several factors, such as age, sex, education level, depressive symptoms, and antidepressant use, their results may indicate that recognition of emotion is mediated by cognitive function.

It is not yet clear which of these perspectives is more accurate. However, these studies suggest that cognitive functions mediate the ability to recognize emotion in elderly people, even considering cognitive tasks other than those used to assess prefrontal function. However, a few points still require clarification. First, Virtanen et al. (2017) used the MMSE as a cognitive task and determined that a decrease of 1 or 2 points was correlated with diminished emotion recognition. The MMSE is a multifactor test that includes immediate memory, orientation, delayed recall, and working memory (Shigemori et al., 2010), and the total MMSE score is calculated as the sum of these abilities. It is not clear which of these abilities may contribute to deterioration of emotion recognition. Second, the prevalence of depression is high in elderly populations and is associated with age-related factors (Djernes, 2006), and depression also affects the recognition of emotions (Demenescu et al., 2010). Therefore, it may be important to consider not only cognitive function but also depressive mood in the emotion recognition paradigm. However, Virtanen et al. (2017) adjusted for depressive symptoms and antidepressant use to calculate the effects of cognitive decline. We believe that depressive mood as well as cognitive function should be considered in analyzing the factors influencing emotion recognition.

The present study was performed to reproduce the effects of aging on the recognition of emotional facial expressions, to clarify the cognitive functions underlying recognition of emotional facial expressions, and to evaluate the effects of depressive mood on recognition of emotion in elderly subjects.

## Materials and Methods

### Participants

We compared 26 adults (12 men, 14 women) aged 19–24 years (mean = 20.9 years) to 30 healthy elderly individuals (15 men, 15 women) aged 61–79 years (mean = 71.6 years). The education level of the older subjects ranged from 9 to 16 years (mean = 13.9 years, standard deviation = 2.2, and median = 14 years). The younger adults were graduate and undergraduate students, with an education level ranging from 13 to 18 years (mean = 15.1 years, standard deviation = 1.2, and median = 15 years). All elderly participants were recruited through the Silver Human Resource Center in Tokyo as paid volunteers. The human resource center was requested to recruit elderly participants conforming to our inclusion criteria, i.e., age from 60s to 70s with no history of dementia or neurological and psychiatric disorders. None of the participants were excluded from the study once it had started.

### Materials

All of the elderly participants were tested using the Japanese version of the neurobehavioral cognitive status examination Cognistat (Matsuda and Nakatani, 2004) to assess their cognitive abilities and the Japanese version of the Geriatric Depression Scale-Short Version (GDS-S-J; Sugishita and Asada, 2009) to evaluate their depressive symptoms.

To evaluate subjects’ emotion recognition abilities, we used emotion matching and emotion selection tasks. We also used face perception and face matching tasks to clarify subjects’ basic face perception ability. The procedures for these four tasks were adopted from previous studies (Miller et al., 2012; Kumfor et al., 2014). The original stimuli were based on Caucasian faces; however, in the present study, to make the stimuli appropriate for a Japanese population, face stimuli were selected from a Japanese facial expression database (Fujimura and Umemura, 2018), including eight models (four males and four females) and six basic emotions (happiness, anger, disgust, sadness, fear, and surprise) and neutral. Pictures were cropped to create long oval shapes to hide the hair and then converted into grayscale images.

#### Face Perception Task

The aim of this task was to confirm subjects’ basic perceptual ability. Each picture was 75.3 × 51.8 pixels (height × width) in size, and the distance between stimuli was 44.8 pixels. A pair of different models or two pictures of the same model, each with the same emotional expression (neutral face), were presented on the computer screen, and subjects were asked, “Are these pictures the same?” We used eight models (four male and four female). There were 28 trials including 20 pairs (8 matched and 12 unmatched pairs). The hit rate (number of correct identifications of a pair/28 pairs) was used as the measure of participants’ performance; the maximum score was 1.0.

#### Face Matching Task

The aim of this task was to clarify subjects’ face identification ability independent of their emotional expression recognition ability. A pair of faces expressing different emotions (six facial expressions and one neutral expression) showing the same model or different models were presented on the computer screen, and subjects were asked, “Do these show the same person?” There were 42 trials (21 matched and 21 unmatched models) and no counterbalancing. We used three male models, and different facial expressions were presented for each trial. The hit rate (number of correct identifications of a pair/42 pairs) was used as the measure of each participant’s performance; the maximum score was 1.0. All stimulus properties in the face matching task were the same as those in the face perception task.

#### Emotion Matching Task

The aim of this task was to evaluate subjects’ emotion identification ability without verbal labeling, independent of face identification. Two faces of different people with the same or different emotional expressions were presented on the computer screen, and the subjects were asked to decide whether each facial expression pair was the same. There were 42 trials (21 matched and 21 unmatched facial expressions) composed of different models and without counterbalancing. We used three male models and presented different models for each trial. The hit rate (number of correct identifications of each pair/42 pairs) was used as a measure of each participant’s performance; the maximum score was 1.0. All stimulus properties of the face matching task were the same as those in the face perception task.

#### Emotion Selection Task

This task measured the participants’ ability to select a facial expression based on a verbal label. The subjects viewed arrays of seven pictures of a single model displaying six emotional expressions and one neutral expression, and they were asked to point to the target face corresponding to a label spoken by the examiner. The size of each picture was 32.2 × 20.6 pixels (height × width). One of the target faces was plotted in the center of the screen, and the other six target faces were plotted at radial distances 48.9 pixels from the center. There were 42 trials, and we used six models (three male and three female). The faces and the positions of the emotions on the screen were varied across trials. To maximize the intrinsic value of the stimuli, a partial credit score was adopted. In this scoring method, each response was given credit based on the proportion of subjects in the reference group giving that response. For example, if a given stimulus was classified as “happy” by 50% of the reference group, “angry” by 40%, and “neutral” by 10%, then the response “happy” would receive a score of 1.0 (0.5/0.5), “angry” would receive 0.8 (0.4/0.5), and “neutral” would receive 0.2 (0.1/0.5). All other responses would receive a score of 0 (Heberlein et al., 2004).

### Procedure

The experiment was conducted in a quiet room with one subject at a time. Before the experimental task, elderly subjects were administered the Cognistat examination and the Japanese version of the GDS-S-J. During the experimental task, the participants were seated 55 cm from the monitor (Iiyama ProLite XU2390HS-B2, Japan). The experimental order of the four tasks was counterbalanced among subjects.

### Statistical Analyses

All statistical analyses were performed using R-3.5.1 (R Core Team, 2018) with the effect size package (Ben-Shachar et al., 2020) for effect size (*d*) analyses, the anovakun package version 4.8.4 (Iseki, 2019) for mixed-model univariate ANOVA, and the MASS package (Fox and Weisberg, 2019) for linear regression analyses. The face perception, face matching, and emotion matching tasks were analyzed using Welch’s *t* test, and the face selection task was analyzed using mixed-model univariate ANOVA, with emotion (anger, disgust, fear, sadness, surprise, happiness, and neutral) as the within-subject variable and age (young, elderly) as the between-subject variable. *Post hoc* analyses using Holm’s sequentially rejective Bonferroni procedure for multiple comparisons were conducted to examine interaction and main effects. In the elderly population, to clarify the effects of emotion recognition ability, stepwise multiple linear regression analyses were conducted. Scores on the face matching and emotion matching tasks were entered as dependent variables, and age, face perception task score, GDS-S-J score, and Cognistat subtest scores were entered as independent variables in separate regression analyses. In all analyses, *P* < 0.05 was taken to indicate statistical significance.

### Ethics

The study design was approved by the Ethical Review Committee of Chuo University Institute of Cultural Sciences (protocol number: 03 [FY2018]). All participants provided written informed consent to participate in the study. Although none of the procedures placed a high burden on the participants, they were told that they could take a rest at any time during the study if necessary.

## Results

Three subjects in the control group were excluded from the analyses due to missing data. Thus, data of 23 young subjects were analyzed (11 men and 12 women, aged 19–24 years [mean = 21.1 years]; Table 1). In addition, due to a ceiling effect, the orientation Cognistat subtest score was excluded from the regression analyses.

**TABLE 1:** Participants’ demographic data.

	**Elderly group**				**Younger group**	***P*-value**
Sex (male/female)	15/15				11/12	*n.s.*^a^
Age (range)	71.6 (61–79)				21.1 (19–24)	<0.001^b^
Education level (range)	13.9 (9–16)				15.1 (13–18)	<0.05^b^

	**Mean (SD)**	**Range**	**Cutoff point**	**Below/above cutoff point (%)**		

GDS-S-J	2.1 (2.4)	0–9	5/6^c^ 9/12^d^	2 (7) 0 (0)		
Cognistat						
Total score	95.6 (5.1)	83–104	82/84	0 (0)		
Orientation	11.9 (0.4)	10–12	10/11	1 (3)		
Attention	7.2 (1.3)	4–8	7/8	10 (33)		
Comprehension	5.8 (0.5)	4–6	5/6	5 (17)		
Repetition	11.3 (1.6)	5–12	9/10	4 (13)		
Naming	7.7 (0.5)	6–8	6/7	1 (3)		
Construction	5.7 (0.6)	4–6	4/5	3 (10)		
Memory	7.8 (2.9)	2–12	8/9	14 (47)		
Calculation	3.9 (0.3)	3–4	3/4	3 (10)		
Similarity	5.6 (1.3)	2–8	3/4	2 (7)		
Judgment	4.0 (1.4)	2–6	2/3	6 (20)		

*^*a*^Pearson’s χ^2^ test; ^*b*^Welch’s *t* test; ^*c*^for depressive tendency; ^*d*^for depressive state.*

### Face and Emotion Tasks

**FIGURE 1:**
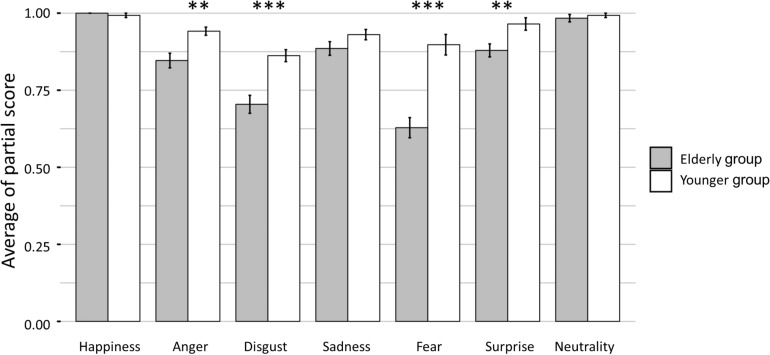
Comparison of the emotion selection task results between the elderly subjects and young subjects. The performance of the elderly subjects was lower than that of young subjects in recognizing facial expressions denoting anger, disgust, fear, and surprise (^∗∗^*P* < 0.01 and ^∗∗∗^*P* < 0.001).

Although there were no significant differences between the two groups in the face perception task [Welch’s *t* (40.8) = –2.0, *P* = 0.057, and *d* = –0.61], significant differences were observed for the face matching [Welch’s *t* (41.0) = –4.5, *P* < 0.001, and *d* = –1.4], and emotion matching tasks [Welch’s *t* (50.8) = –2.4, *P* = 0.02, and *d* = –0.7]. These results indicated that young and elderly subjects had similar performance in face perception, whereas elderly subjects had lower face identification and emotion recognition abilities. In addition, the emotion selection task showed a significant interaction [*F* (3.9) = 10.2, *P* < 0.001, and η*^2^_*g*_* = 0.14], and the single main effect tests revealed that the performance in emotional face recognition was lower in the elderly group than in young adults for the emotions of anger, disgust, fear, and surprise (Figure 1).

### Multiple Regression Analyses in the Elderly Group

Multiple regression analyses were conducted to identify the predictors of emotion recognition ability in the elderly group. Explanatory variables were selected by stepwise selection using Akaike’s information criterion. We calculated the variance of inflation factor (VIF) for the explanatory variables of the input candidates, and found that the value ranged from 1.2 to 2.4 and no VIF score exceeded 10, which were considered to cause multicollinearity (Belsley et al., 1980). In the face matching task, the *F*-value of the model was significant, although the adjusted *R*^2^ was low (_adj_*R*^2^ = 0.21, *P* < 0.05); however, the adjusted coefficient of multiple determination (*R*^2^) for the emotion matching task was significant (_adj_*R*^2^ = 0.71, *P* < 0.001). Partial regression coefficients for each factor are shown in Table 2.

**TABLE 2:** Predictors of face matching and emotion matching tasks.

	**Face matching task**		**Emotion matching task**	
	***B***	**β**	***B***	**β**
Age			–0.97	−0.86***
GDS–S–J			–1.14	−0.57***
Face perception task			0.57	0.46***
Cognistat				
Attention	2.62	0.25		
Comprehension	6.70	0.25	1.58	0.16
Repetition			–1.53	−0.52***
Naming			2.45	0.27*
Construction				
Memory				
Calculation	–16.52	–0.38		
Similarity	3.96	0.40*		
Judgment			0.52	0.15
Adjusted *R*^2^	0.21		0.71	
*F*	2.97*		11.14***	

*P < 0.05, and ***P < 0.001.

## Discussion

This study was performed to reproduce the effects of aging on the recognition of emotional facial expressions, to clarify the cognitive functions underlying recognition of emotional facial expressions, and to evaluate the effects of depressive mood on recognition of emotion in elderly subjects. To achieve these aims, we compared experimental emotion recognition tasks and a multicomponent neuropsychological test instead of using a simple screening test (Virtanen et al., 2017). The results were as follows. First, compared to young adult subjects, elderly subjects showed clear deterioration of their ability to recognize emotional facial expressions. Notably, the effects of age varied among categories of emotional expression. Second, regression analyses indicated that performance on the emotion matching task was predicted by depressive mood, cognitive function, and age.

Previous studies have reported that cognition of negative facial expressions, such as anger and fear, declines with age, while cognition of positive facial expressions, such as joy, is less affected by aging (Calder et al., 2003; Suzuki et al., 2007). Age-related changes and their discrepancies between categories have often been explained as positivity bias supporting socioemotional selectivity theory (Mather and Carstensen, 2005). That is, it is believed that elderly people act and think in pursuit of current happiness rather than information that will be beneficial in the future. The results of the present study also replicated these phenomena in elderly participants. In addition, age-related changes may be related to the study paradigm used. Suzuki and Akiyama (2013) reported that when neutral expressions were presented momentarily followed by labeling of the presented expressions, the hit rate of joyful expressions decreased and false positives increased in elderly participants. On the other hand, Saito et al. (2020) reported that immediate detection of joyful expressions by the visual search paradigm is less likely to occur in elderly participants. In the present study, seven different facial expressions were presented at once suggesting that happiness was relatively easy to identify.

As reported previously (Keightley et al., 2006; Mill et al., 2009) we confirmed that older participants had poor ability to recognize emotional facial expressions. In addition, our study confirmed that face recognition ability declined not only during tasks with a high cognitive load, such as in the emotion selection task, which required participants to select a facial expression based on a verbal label, but also during low cognitive load tasks, such as the emotion matching task, in which subjects were instructed to match emotional expression stimuli. These results suggested that the decline in emotional expression recognition in elderly people is a universal phenomenon independent of task demands. In addition, our study demonstrated that an aging effect was apparent not only compared to young subjects but also in comparisons among elderly subjects. In multiple regression analyses, age was a powerful predictor of performance in the emotion matching task. Therefore, aging may be a crucial factor in the decline in ability to recognize emotional facial expressions. In contrast, in the face matching task, which showed significant declines compared to performance among younger subjects, the factor of aging was not a predictor of face identification among elderly subjects of different ages. These results suggested that, compared to the face matching task, the emotion matching task was relatively sensitive to aging in old age.

In multiple regression analyses, our study also confirmed that depressive mood was related to performance on the emotion matching task. Although Demenescu et al. (2010) reported impaired recognition of facial expressions in subjects with anxiety and major depression, their subjects were diagnosed patients, whereas our subjects had not been diagnosed. The cutoff points for the GDS-S-J are 5/6 for depressive tendency and 9/12 for depressive state (Watanabe and Imagawa, 2013); in the present study, only two subjects exceeded the cutoff for depressed mood, and none demonstrated a depressed state. These results suggested that not only clinical depression but also emotional state within the normal range may be crucial for recognition of facial expressions.

Demented patients have also been reported to show a decline in the recognition of facial expressions, and a relationship between this recognition deficit and cognitive capacities has been demonstrated (Torres Mendonça De Melo Fádel et al., 2019). In the present study, however, the elderly participants did not have dementia; rather, our elderly subjects were recruited from a community job service, and all scored above the cutoff score on the Cognistat. These results suggest that the decline in emotion recognition may be related to aging, and not to pathological changes in the brain. However, our elderly subjects may not have been completely healthy subjects. Some showed decreased scores on Cognistat subtests, with the most notable decline being on the memory subtest followed by the attention (digit span) subtest; 47% (14/30) and 33% (10/30) of elderly subjects, respectively, were below the cutoff points in these subtests. Therefore, our subjects may have included some individuals from a preclinical population, such as people with mild cognitive impairment (MCI; Petersen et al., 1999). At the time of this experiment, our elderly subjects had no apparent issues related to their cognitive function, and their functional abilities were apparently preserved (i.e., they could manage their schedule and come to the experimental room independently). However, some showed declines in one or more cognitive domains. Therefore, some of our subjects may have met the diagnostic criteria for MCI (Albert et al., 2011) or mild cognitive disorder (American Psychiatric Association, 2013). We cannot define a biological substrate related to age at this stage, so MCI should be taken into consideration in explaining the effects of aging on the recognition of emotional expression.

The present study revealed an effect of hidden cognitive decline and depressive mood on recognition of facial expression within an elderly population. However, our study had limitations. First, the sample size and deviation of cognitive function test are small in the elderly, and therefore we could not conclude that cognitive functions and depressive mood definitely affect recognition of emotional facial expressions. Second, we did not examine cognitive function and depressive scale in young adults, so the predictors of facial cognition shown in this study may have been limited to the elderly. Third, the face recognition paradigm used was different from that used by Virtanen et al. (2017), and therefore it remains possible that differences between the results of these studies may have been due to differences in the paradigms used.

In conclusion, we found that elderly individuals showed a decline in the recognition of emotional facial expressions. The effect of age was apparent not only compared to a young population but also relative to older subjects. In addition to the effect of age, emotional status (depressive tendencies) and cognitive function were also related to recognition of emotional expression, but paradoxical relationships were apparent between recognition of emotional faces and some verbal functions. It may be important to consider preclinical pathological changes, such as MCI, in evaluating the effects of age in elderly populations.

## Data Availability Statement

The data analyzed in this study is subject to the following licenses/restrictions: The data are not publicly available due to ethical restrictions. The data that support the findings of this study are available on request from the corresponding author, AM. Requests to access these datasets should be directed to AM, green@tamacc.chuo-u.ac.jp.

## Ethics Statement

The studies involving human participants were reviewed and approved by Ethical review committee of Chuo University Institute of Cultural Sciences [protocol number: 03 (FY2018)]. The participants provided their written informed consent to participate in this study.

## Author Contributions

AM and RO designed experiments. RO performed the experiments. RO analyzed the results. AM and RO wrote the manuscript. Both authors read and approved the final manuscript.

## Conflict of Interest

The authors declare that the research was conducted in the absence of any commercial or financial relationships that could be construed as a potential conflict of interest.
